# Evolution of Tuberculosis/Human Immunodeficiency Virus Services among Different Integrated Models in Myanmar: A Health Services Review

**DOI:** 10.3390/tropicalmed4010002

**Published:** 2018-12-24

**Authors:** Myo Su Kyi, Si Thu Aung, Edward McNeil, Virasakdi Chongsuvivatwong

**Affiliations:** 1 Regional Public Health Department, Nay Pyi Taw Union Territory 15011, Myanmar; 2 Epidemiology Unit, Faculty of Medicine, Prince of Songkla University, Hat Yai, Songkhla 90110, Thailand; edward.m@psu.ac.th (E.M.); cvirasak@medicine.psu.ac.th (V.C.); 3 National Tuberculosis Programme, Ministry of Health and Sports, Nay Pyi Taw Union Territory 15011, Myanmar; dr.sta.ntp@gmail.com

**Keywords:** tuberculosis/human immunodeficiency virus, integrated services, ART, Myanmar

## Abstract

Myanmar is one of the highly affected countries by tuberculosis (TB) and human immunodeficiency virus (HIV) co-infection. We aimed to review the coverage of TB/HIV integrated services as well as to document the performance of this integrated services. A retrospective program review was conducted using the aggregated data of the National TB Programme (NTP) from 2005 to 2016. In Myanmar, TB/HIV services were initiated in seven townships in 2005. Townships were slowly expanded until 2013. After that, the momentum was increased by increasing the government budget allocation for NTP. In 2016, the whole country was eventually covered by TB/HIV services in different types of integration. Antiretroviral therapy (ART) coverage among HIV-positive TB patients remained low and it was the only significant difference among the three types of integration. Barriers of low ART coverage need to be investigated to reduce the burden of TB/HIV.

## 1. Introduction

Myanmar, a country located in South-East Asia, is one of the 30 high tuberculosis (TB) burden countries with a prevalence (bacteriologically positive TB) of 434/100,000 population according to the National TB Prevalence Survey in 2010 [1]. The prevalence of human immunodeficiency virus (HIV) in some areas of Myanmar is among the highest in the Asia-Pacific region and the estimated HIV prevalence at the national level was 0.59% among those aged 15 years or more in 2015 [2]. Consequently, Myanmar has become one of the countries mostly affected by TB/HIV co-infection. In 2016, of 139,625 TB patients notified, 10,952 were living with HIV [3].

To alleviate the dual burden of TB/HIV in populations at risk, the World Health Organization (WHO) developed policies and guidelines for TB/HIV services [4]. Integration of TB/HIV services improve the health outcomes of TB/HIV co-infected patients with the benefits of timely ART uptake [5,6,7,8,9,10], increased uptake of HIV testing and CPT [7,11,12,13,14] and better TB treatment outcomes [7,12,14,15,16,17]. Although it is recommended to extend further integration of services, the two programs for TB and HIV in most countries remain separated with their own implementation strategies and funding flows. Thus, the integration of these two programs encounters challenges from policy making to integration at the implementation level [18].

In Myanmar, the National TB Programme (NTP) and National AIDS Programme (NAP) are vertical programs under the Ministry of Health and Sports. In 2016, the NTP was operating with 14 regional/state TB centers and 101 TB teams at district and township levels. The whole country has been covered with Directly Observed Treatment, Short Course (DOTS) since 2003. The NAP was operating with 113 antiretroviral therapy (ART) centers and 140 decentralized ART clinics across the country. ART started in 2005 but up until now does not cover the whole country. 

TB and HIV control activities in Myanmar are supported by the government along with a number of international donor organizations [19], the largest support coming from the Global Fund to fight AIDS, Tuberculosis and Malaria (GFATM) [2,20]. In 2013, the GFATM launched a new funding model that served as an instrument towards identifying key populations, developing the single proposal (concept note) for TB and HIV to enhance joint programming and integrated implementation of activities [21].

Following the WHO policy, TB/HIV collaborative services have been implemented since 2005 in Myanmar. There is thus a need to review the progress of TB/HIV services over the past decade. The objectives of this study are to review the coverage of TB/HIV integrated services and to document the performance of TB/HIV services in Myanmar.

## 2. Materials and Methods 

### 2.1. Study Design and Data Sources

A retrospective program review was conducted using the aggregated data extracted from routine surveillance reports of the NTP. Table 1 defines the WHO recommended core indicators representing the performance of activities which are implemented by the NTP [22,23]. Our study used these three core indicators as a conventional assessment of the performance of TB/HIV services.

### 2.2. Data Analysis

To describe the changes over time, trends were fitted on a logarithmic scale due to the highly skewed distribution of the data. A Joinpoint regression model was used to estimate the regression coefficients with a joinpoint between the government budget allocation and TB/HIV township expansion. Joinpoint regression model is a well-known approach used to study varying trends over time that identifies the time point(s) in which the trend significantly changes, and estimates the regression function with joinpoint(s) previously identified [24,25]. The performance of TB/HIV services are summarized using the median and interquartile range (IQR). The Pearson product–moment correlation coefficient was calculated to assess the strength of linear association between the proportions of each core performance indicator. The Kruskal–Wallis test was used to quantify the difference in the performance of each type of integration with significance level set at 0.05. All analyses were conducted using R version 3.5.1 (https://cran.r-project.org).

For trend analysis, we used the compiled data of government and non-government sectors. For analysis of performance among different types of integration in the year when the whole country was covered by TB/HIV services, we used the data of government sector only because the nature of the services provided by the non-government sector was not well identified.

### 2.3. Ethics 

The study was approved by the Ethical Review Committees from Prince of Songkla University, Thailand (REC Number: 59-278-18-5) and the Department of Medical Research, Myanmar.

## 3. Results

### 3.1. Evolution of TB/HIV Services

In Myanmar, the Central Coordinating Body for TB/HIV services was organized in 2005 and strengthened in 2012 with the guidance of the Ministry. This coordinating body was established at state/regional, district and township levels to be functioning according to the scale-up plan of TB/HIV townships. TB/HIV services were initiated in seven townships in 2005 and expanded to 136, 236, and 330 townships by 2014, 2015, and 2016, respectively. 

In townships which implement the fully integrated model, TB and HIV services are provided at township hospitals managed by the township medical officers. A partially integrated model is provided using two approaches. One approach is that both vertical teams coexist in the same township, referring cases to each other for the services not available in the referring center. The second approach is to have only TB services; patients with HIV are referred to an HIV clinic in the nearest township.

**Figure 1:**
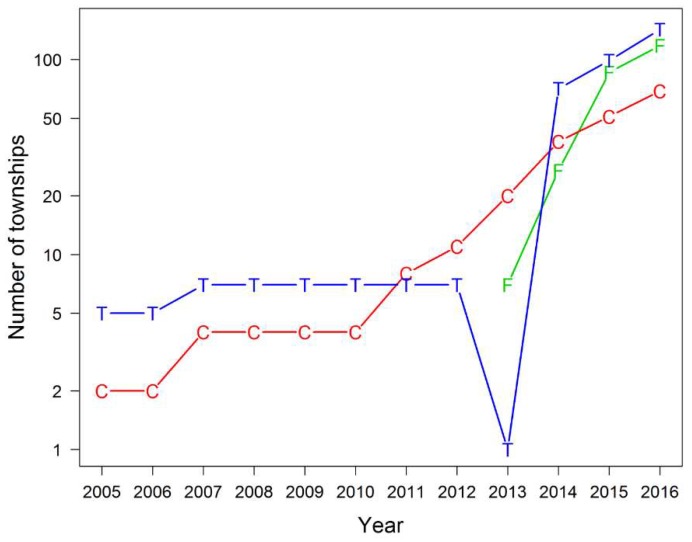
Number of townships in different types of integrated TB/HIV services (2005–2016). F = fully integrated model; C = partially integrated model: TB and HIV services co-existed; T = partially integrated model: TB services only.

Figure 1 demonstrates the number of townships in different types of integration from 2005 to 2016. Partially integrated model in both approaches were initiated in 2005. In 2013, the fully integrated model was introduced and all seven townships that were previously classified as using the partially integrated model with only TB services became fully integrated townships. Between 2013 and 2016, all three groups showed an increase in the number. 

**Figure 2:**
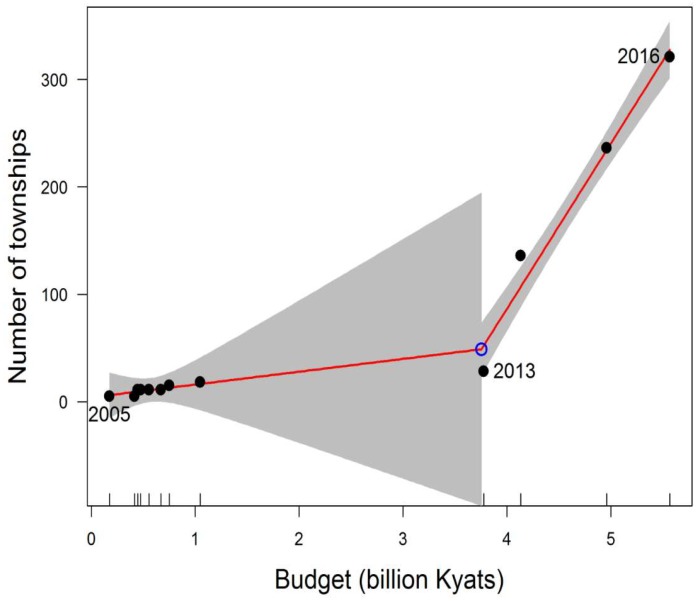
Relationship between government budget allocation for National TB Programme (NTP) and number of TB/HIV townships (2005–2016) using Joinpoint regression. Note: The grey shading represents 95% confidence interval of the estimated value.

Figure 2 shows the relationship between government budget allocation for the NTP and total number of townships with TB/HIV services between 2003 and 2016. The relationship is evident from the joinpoint regression showing two clear periods with vastly differing slopes with a breakpoint at 2013. During the first period (before 2013), the relationship was not significant. Since 2013, there was an average of 1.4 townships established for every 10 million kyats allocated from the budget.

### 3.2. Performance of TB/HIV Services

TB/HIV services which are provided at different integrated models in Myanmar are summarized in Table 2. Items of services are described in the first column. Under a fully integrated model of care, the same clinic offers all services for TB and HIV patients (second column). Under a partially integrated model of care, TB clinics (third column) offer all services related to TB and HIV counseling and testing service but refer those with HIV co-infection to an HIV clinic for ART and cotrimoxazole preventive therapy (CPT). HIV clinics (last column) offer all care and supports for people living with HIV, including TB symptom screening and provision of IPT (isoniazid preventive therapy), but refer those presumptive of TB to TB clinics for further necessary management. A cross-referral system between the NTP and NAP has been developed and the recording and reporting framework is standardized. Both TB and HIV programmes use patient cards as the data source for paper-based registers. TB and HIV clinics separately report summaries of TB/HIV collaborative services to the respective programmes at the central level where data are compiled quarterly in an electronic system.

**Figure 3:**
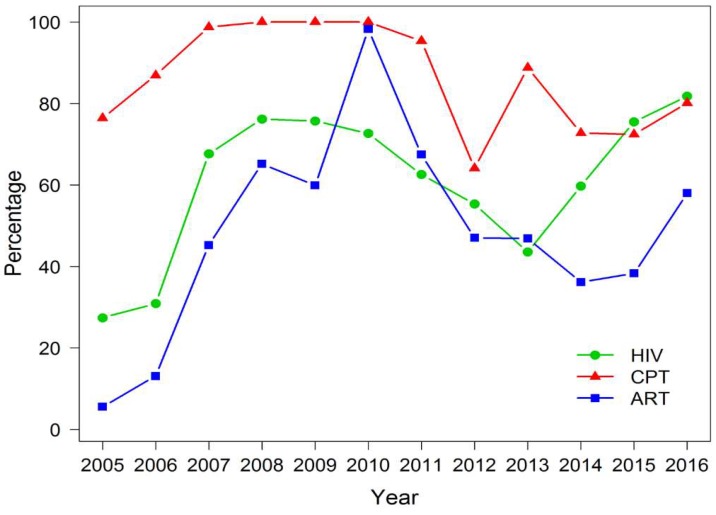
Performance of TB/HIV services including government and non-government sector (2005–2016). HIV: TB patients with known HIV status; CPT: HIV-positive TB patients who received CPT; ART: HIV-positive TB patients who received ART.

Figure 3 shows the conventional assessment of the performance of TB/HIV services from 2005 to 2016. The trends of all services varied across years. The percentage of TB patients with known HIV status markedly increased from 27% (95% CI: 25.2–29.7) in 2005 to 82% (95% CI: 81.6–82.0) in 2016. Similarly, the proportion of HIV-positive TB patients who received ART has markedly increased from 6% (95% CI: 1.5–13.6) in 2005 to 58% (95% CI: 57.1–59.0) in 2016. All rates decreased between 2011 and 2014. 

**Figure 4:**
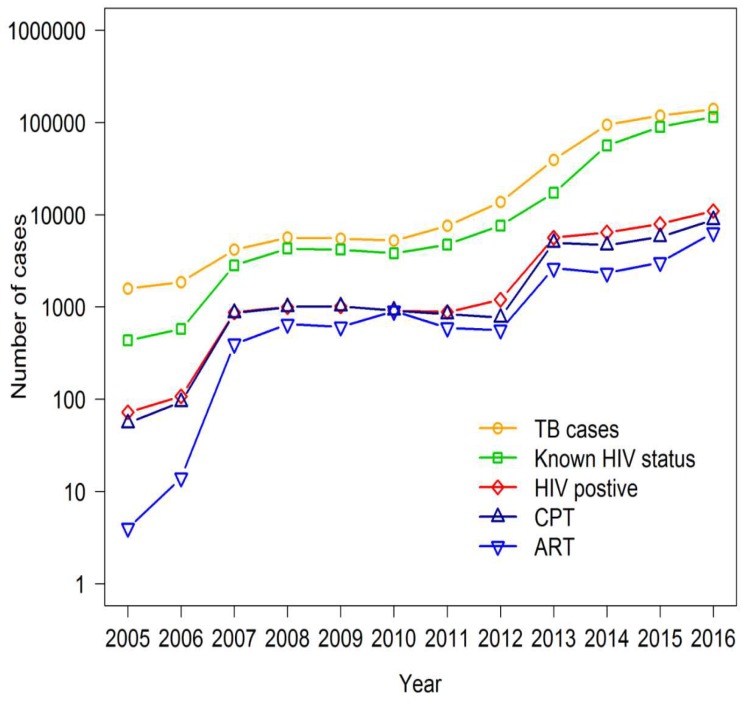
Trends of TB/HIV service cascade in number of cases (2005–2016). Note: Data included government and non-government sector. CPT: HIV-positive TB patients who received CPT; ART: HIV-positive TB patients who received ART.

Because this type of performance monitoring might give misleading information, Figure 4 describes the trends of TB/HIV service cascade in a logarithmic scale from 2005 to 2016. The number of registered TB cases increased each year, of which cases with known HIV status also increased. The narrowest gap between these two trends was observed in 2016. Among TB patients with known HIV status, the proportion of HIV-positive cases eventually reduced in 2016 as the gap between the two series widened. The gap between HIV-positive TB cases and those who received CPT consistently narrowed while the gap between HIV positive TB cases who received ART were initially wide and eventually narrowed.

**Figure 5:**
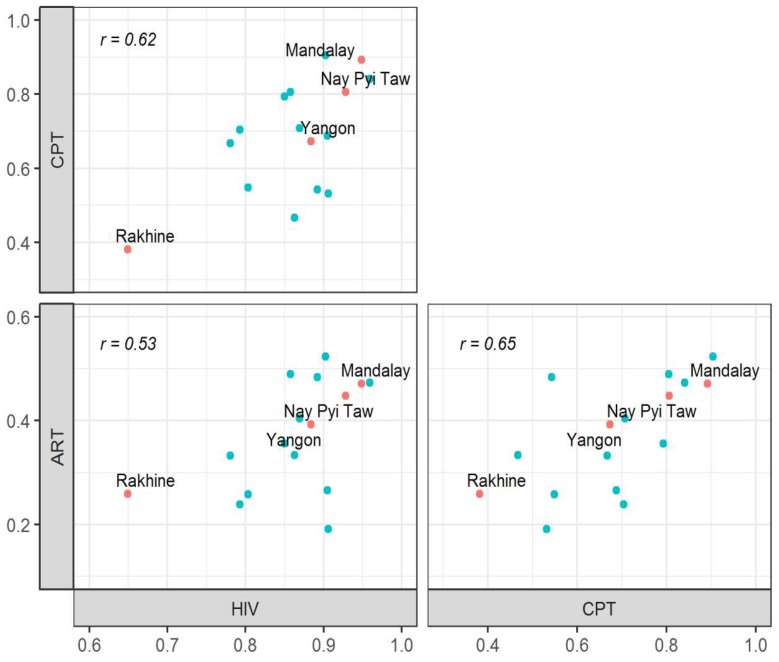
Correlations between the three recommended core performance indicators for TB/HIV services in the 15 states/regions of Myanmar in 2016. Note: Data included only government sector. The highlighted points correspond to regions with low performance (Rakhine) or high development. HIV: TB patients with known HIV status; CPT: HIV-positive TB patients who received CPT; ART: HIV-positive TB patients who received ART; *r*: Pearson product–moment correlation coefficient.

An evaluation of the performance of TB/HIV services according to states/regions in 2016, when the coverage in the whole country was 100%, is shown in Figure 5. The proportions of all three services were significantly correlated with each other, with the Pearson product–moment correlation coefficient, between HIV testing and CPT of 0.62, between HIV testing and ART of 0.53, and between CPT and ART of 0.65. Low rates of services were observed consistently in Rakhine State while high rates were observed in Yangon, Mandalay and Nay Pyi Taw Regions.

Table 3 shows a comparison of the performance of TB/HIV services by the three different integrated models in 2016. The only significant difference was observed on the percentage of HIV-positive TB patients who received ART, which was more common in the partially integrated model with TB and HIV services co-existed (Kruskal–Wallis test, *p* value = 0.02). However, ART coverage among TB/HIV co-infected patients was low across all types of integration, accounting for less than 50%. 

## 4. Discussion

We conducted a study in Myanmar with the aims to review the coverage of TB/HIV integrated services and to document the performance of TB/HIV services. In Myanmar, TB/HIV services were initiated in seven townships in 2005 and the whole country was covered by 2016, 11 years later. The performance of TB/HIV services was found to increase over these 11 years. The proportion of HIV-positive TB patients who received ART was low across all models. In 2016, Rakhine State had the lowest rates of all services, whilst Yangon, Mandalay and Nay Pyi Taw achieved the high rates.

Townships for TB/HIV services were slowly expanded until 2013 without significantly associated with the government budget allocation for NTP. After that, the momentum was increased by increasing budget allocation. This finding may have more explanations. TB/HIV townships expansion did not depend solely on the expenditure of the NTP. Because of two vertical programs’ collaboration for TB/HIV services, it relied also on the NAP side, especially for provision of ART. The NAP introduced decentralized ART clinics at township level hospitals in 2013 and then expanded geographically [2]. Furthermore, the new funding model of the GFATM, initiated in 2013, may have contributed to that achievement. With similar explanations, the fully integrated model could be started in 2013.

On assessment of the performance based on the proportions of cases adhering to the WHO core performance indicators for each of the services, there was a period when all services appeared to show a decline. However, after viewing the trends on a logarithmic scale, the performance of all services was satisfactory throughout the 12 year period. This suggests that using the proportions of services to assess the performance over time may be invalid, especially at the time when services are being expanded, although it is useful as indicators for assessment of target achievement. 

ART coverage represents the level of HIV care and support to TB/HIV patients. WHO recommends that ART should be provided for all HIV-positive TB patients [4]. Globally, about 85% of notified HIV-positive TB patients were reported to be on ART in 2016 [26]. However, in our study, the highest rate of ART coverage, observed in 2016, was only 58%. ART coverage was less than 50% across all types of integration on analysis of the data from only government sector in 2016. It might be due to the limitation which did not include the data from non-government sector that contributed about 19% of all TB notifications in 2016 [3]. The HIV testing rate was enhanced and the achievement in 2016 (82%) was appreciable compared with the global rate (57%) [26]. In this study, CPT coverage was the best among all services. 

In 2016 when the services started to cover the whole country, Rakhine State was observed to have the lowest performance across all services. Rakhine State is located in the west of Myanmar bordering Bangladesh. A series of violent clashes have been ongoing for many years which have subsequently affected the health care services in Rakhine State. The most developed regions, such as Yangon, Mandalay and Nay Pyi Taw, had high performances across all services. ART coverage was observed the highest in the partially integrated townships with both vertical teams, possibly due to the higher level of expertise and capacity of the clinic staff to manage patients with TB and HIV. Another explanation might be due to HIV patients having easy access to ART services because the townships are regional/state or district level townships.

Fully integrated model is likely to benefit patients by having lower transportation costs if visits for TB and HIV are at the same clinic on the same day [27] and reduce the referral problems between TB and HIV clinics [28]. However, it might require the additional resources and expertise of staff for implementation of fully integrated model [27]. Besides, TB transmission is a risk at the place where both TB and HIV patients attend [29,30]. Our study has limited information on challenges and opportunities of different integration services. This conjecture needs to be evidenced by further studies. 

## 5. Conclusions

Myanmar could expand well TB/HIV services. The performance of HIV testing and CPT provision was adequate. However, ART coverage among HIV-positive TB patients remained low across all types of integration. Barriers of low ART coverage need to be investigated to reduce the burden of TB/HIV.

## Figures and Tables

**Table 1:** WHO recommended core indicators for performance of tuberculosis/human immunodeficiency virus (TB/HIV) services. CPT: cotrimoxazole preventive therapy.

Core Indicator	Numerator	Denominator
Proportion of TB patients with known HIV status	Number of TB patients registered during the reporting period that had a HIV test result and recorded in the TB register.	Total number of TB patients registered during the reporting period.
Proportion of HIV-positive TB patients who received CPT	Number of HIV-positive TB patients, registered over the reporting period, starting or continuing CPT treatment during their TB treatment.	Total number of HIV positive TB patients registered during the reporting period.
Proportion of HIV-positive TB patients who received ART	Number of HIV-positive TB patients registered over the reporting period starting or continuing ART treatment during their TB treatment.	Total number of HIV positive TB patients registered during the reporting period.

**Table 2:** Availability of Services for TB/HIV co-infected patients.

Services	Fully Integrated Model (118 †)	Partially Integrated Model (212 †)	
		TB Clinics	HIV Clinics
**HIV services**			
HIV counseling and testing	+	+	+
ART provision	+	Refer to HIV clinic	+
CPT provision	+	Refer to HIV clinic	+
**TB services**			
TB symptom screening	+	+	+
Diagnosis and treatment of TB (including Xpert MTB/RIF * assay)	+	+	Refer to TB clinic
IPT provision	+	+	+

† Number of townships with the specific integrated model in 2016; * Mycobacterium tuberculosis/resistance to rifampicin; TB: tuberculosis; HIV: human immunodeficiency virus; ART: antiretroviral therapy; CPT: cotrimoxazole preventive therapy; IPT: isoniazid preventive therapy.

**Table 3:** Median (interquartile range (IQR)) percentage of target patients who received the specific service from government sector by different integrated models in 2016.

Integrated Model				
Partial				
	Full	TB and HIV co-Existed	TB Only	*p* Value †
TB patients with known HIV status	93 (77.9–98.1)	93 (82.5–98.6)	90 (76.3–98.5)	0.63
HIV-positive TB patients who received CPT	81 (50.0–100)	82 (62.2–91.1)	74 (33.3–100)	0.43
HIV-positive TB patients who received ART	32 (19.4–50.0)	45 (28.4–59.0)	33 (8.3–57.1)	0.02

† Kruskal-Wallis test.
